# Catheter Displacement Into the Amniotic Cavity by Fetal Movement After Thoracoamniotic Shunting

**DOI:** 10.7759/cureus.70570

**Published:** 2024-09-30

**Authors:** Daisuke Katsura, Ayako Inatomi, Shinsuke Tokoro, Shunichiro Tsuji, Takashi Murakami

**Affiliations:** 1 Department of Obstetrics and Gynecology, Shiga University of Medical Science, Otsu, JPN

**Keywords:** catheter displacement, fetal movement, fetal pleural effusion, fetal ultrasound, thoracoamniotic shunting

## Abstract

Thoracoamniotic shunting (TAS) is an effective treatment for fetal pleural effusion. We report a case of bilateral fetal pleural effusion in which catheter displacement into the amniotic cavity occurred due to fetal movement after TAS. Initially, left TAS was performed twice using a 60-mm catheter, resulting in displacement and the catheter being pinched between the fetal fingers. Subsequently, a 50-mm catheter was employed for left TAS, which successfully prevented further displacement. Labor was induced at 35 weeks and three days of gestation due to an increase in right fetal pleural effusion. Following delivery, the infant was managed with directional positive airway pressure and drainage of the pleural effusion and remained stable on respiratory support. This case highlights the importance of tailoring catheter length and placement based on the thickness of the fetal chest wall to minimize displacement risks associated with fetal movement.

## Introduction

Fetal pleural effusions, characterized by the accumulation of fluid in the fetal pleural space, can be classified as primary or secondary, with an estimated incidence of 1 in 10,000 to 15,000 pregnancies. Primary effusions are typically chylothorax, which occurs due to leakage of lymphatic fluid resulting from lymphatic maldevelopment, while secondary effusions arise from conditions such as cardiac malformation, fetal anemia or infection, aneuploidy, or structural anomalies that can compress the lungs and mediastinum [1-4]. Smaller effusions may resolve spontaneously or remain stable, whereas larger effusions may lead to pulmonary hypoplasia and fetal hydrops due to circulatory failure. The presence of hydrops has been suggested as an independent predictor of poor outcomes, with a reported survival rate of 31% [2].

Thoracoamniotic shunting (TAS) is a well-established procedure for managing fetal pleural effusion, and the survival rate for fetuses with hydrops following TAS has been reported to be between 57% and 71% [1-4]. This intervention aims to reduce the pleural effusion and alleviate associated complications such as pulmonary hypoplasia and fetal circulatory failure [1-4]. Despite its effectiveness, TAS is not without risks. Common complications include catheter displacement and obstruction, infection, polyhydramnios, and rupture of the fetal membranes [2-4]. Specifically, catheter displacement into the amniotic cavity has been reported in approximately 13.8% of cases, necessitating reintervention [4]. Such displacement not only complicates the procedure but also increases the risk of additional interventions and associated complications. The number of procedures has been reported to be associated with prognosis [4]. Therefore, strategies to prevent catheter displacement are crucial for improving outcomes and minimizing the need for reintervention. We report a case in which we were able to prevent catheter displacement by adjusting the length and placement of the catheter according to the thickness of the fetal chest wall.

## Case presentation

**Figure 1:**
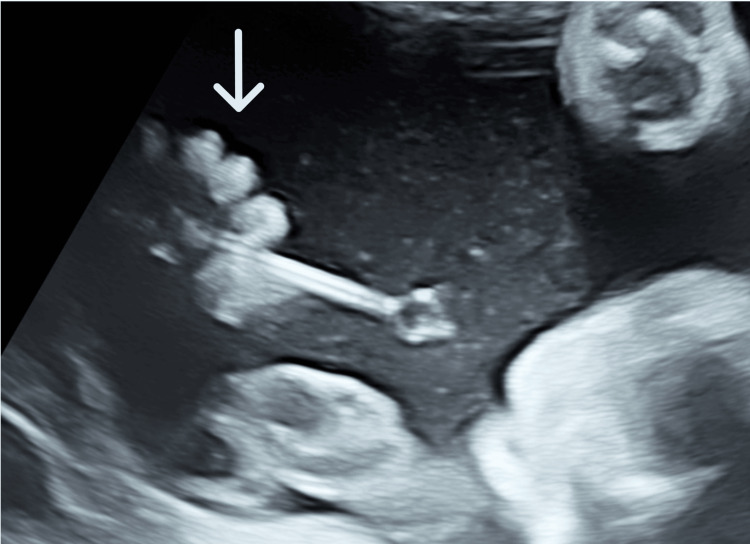
Fetal echography finding The double-basket catheter shunt is pinched between fetal fingers (white arrow).

A 33-year-old woman (gravida 2, para 0) was referred to our hospital for management of fetal bilateral pleural effusions at 31 weeks and five days of gestation. On fetal ultrasound screening, the estimated fetal weight was 2015 g (-1.5 standard deviation), the amniotic fluid index was 21 cm, and bilateral pleural effusion was confirmed, predominantly on the left side, with no structural abnormality. We performed thoracocentesis for the left pleural effusion and diagnosed chylothorax based on a lymphocyte count of 97% in the pleural fluid. At 32 weeks of gestation, we conducted left TAS due to fetal hydrops caused by circulatory failure from massive pleural effusion, as the effusion had reaccumulated and slight ascites was noted two days after the initial thoracocentesis. Although the ascites resolved after TAS, the catheter was displaced into the amniotic cavity due to intense fetal movement. Consequently, left TAS was performed again at 32 weeks and six days of gestation due to the reaccumulation of the effusions. However, the catheter got displaced into the amniotic cavity again and was pinched between the fetal fingers (Figure 1).

**Figure 2:**
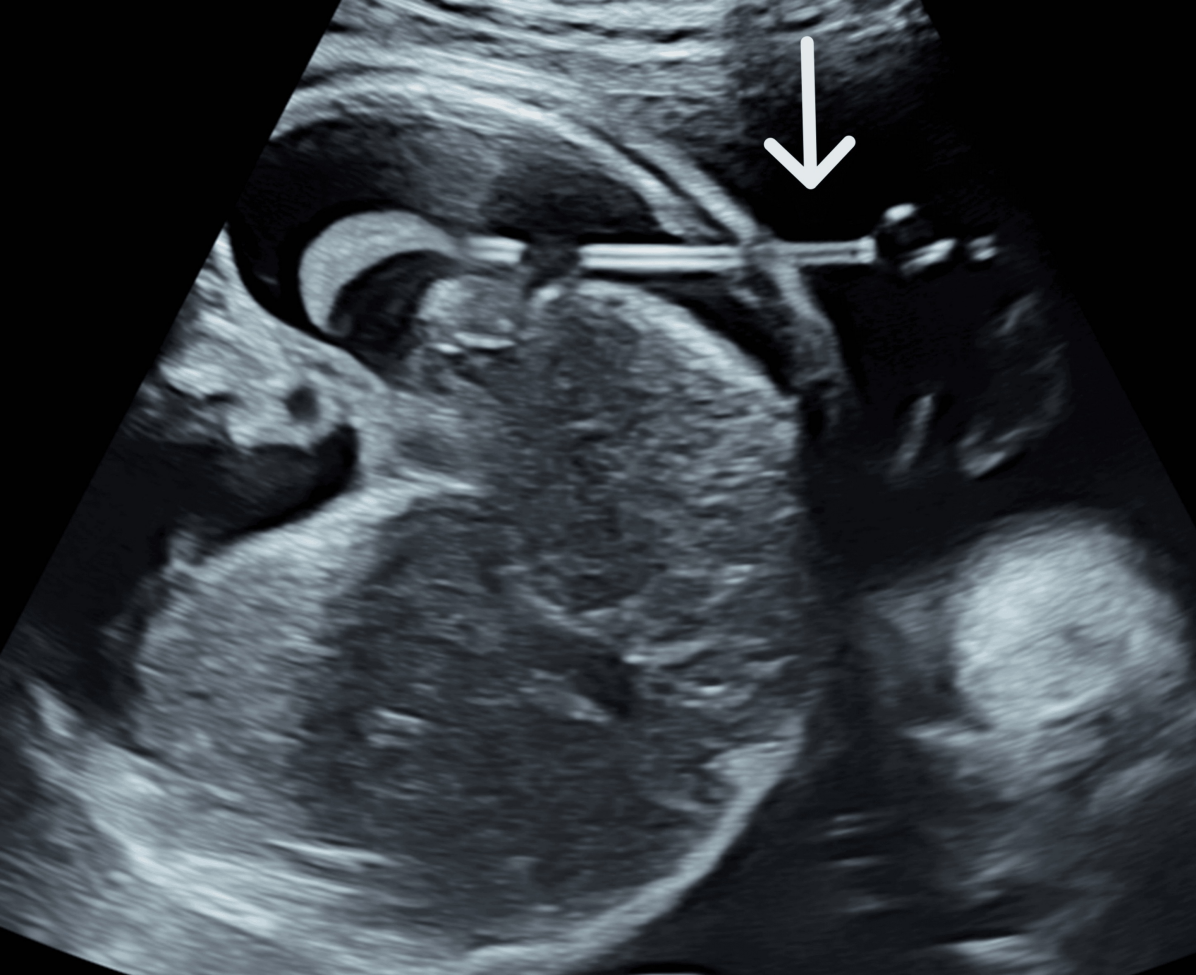
Second fetal echography finding The 60 mm catheter of the double-basket shunt was inserted into the left fetal thoracic cavity (white arrow).

The pleural effusion reaccumulated, and slight ascites was confirmed. We used a 60 mm catheter of a double-basket shunt (Hakko Co., Nagano, JPN) at the first and second TAS (Figure 2).

**Figure 3:**
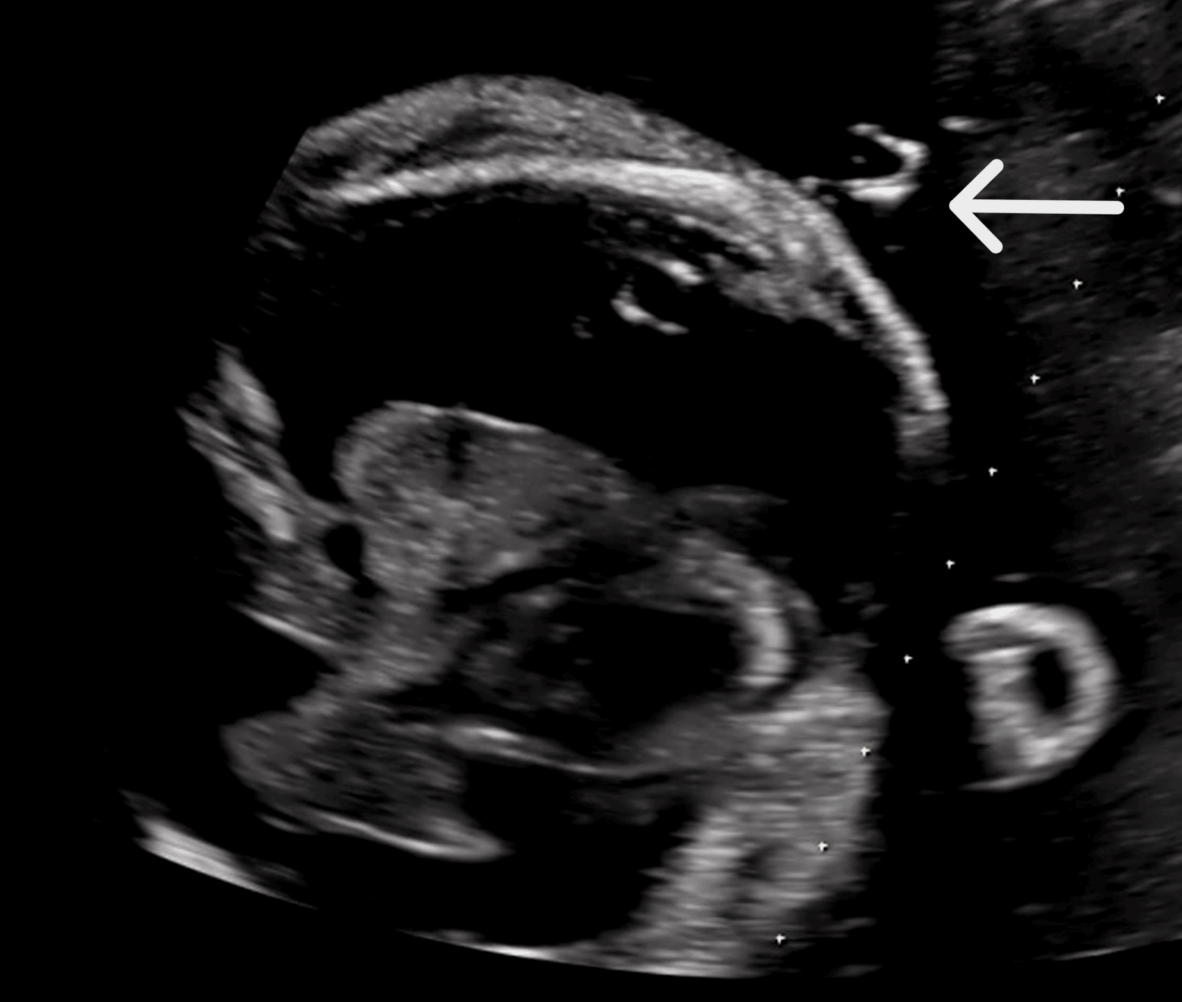
Third fetal echography finding The 50 mm catheter of the double-basket shunt was inserted into the left fetal thoracic cavity (white arrow).

Therefore, we repeated left TAS using a 50 mm catheter at 33 weeks and three days of gestation (Figure 3), and the catheter was not displaced thereafter.

However, we decided to induce labor due to an increase in the right fetal pleural effusion. At 35 weeks and three days of gestation, a male infant weighing 2377 g was born, with an Apgar score of 3 at one minute and 9 at five minutes. His umbilical arterial blood pH was 7.250, and he was admitted to the neonatal intensive care unit. The infant was managed with directional positive airway pressure and drainage of the pleural effusion. He remained stable on respiratory circulation. He was diagnosed with congenital chylothorax, and no chromosomal abnormality was detected. Although medium-chain triglycerides, prednisolone, and octreotide were ineffective for managing the pleural effusions, he responded well to chemical pleurodesis, resulting in the disappearance of the effusions. Following this intervention, his condition remained stable. At three years of age, he demonstrated normal development.

## Discussion

Although TAS is effective for managing fetal pleural effusion, it may be associated with complications [2-4]. Among these complications, catheter displacement occurs frequently and can necessitate reintervention [4]. Therefore, preventing catheter displacement is crucial. In this case, TAS was effective in treating fetal hydrops with ascites due to massive pleural effusion; however, catheter displacement occurred as a complication, requiring reintervention. The catheter was pinched between the fetal fingers, indicating that fetal movement may have contributed to catheter displacement. While definitive proof that the fetus actively displaced the catheter is challenging to establish, this phenomenon suggests that fetal movement could be a significant factor in such displacements.

Reducing the area of the catheter in contact with the fetus might prevent catheter displacement into the amniotic cavity due to fetal movement. It is necessary to shorten the distance between the catheter basket on the amniotic cavity side and the fetal chest wall to reduce the area of the catheter in contact with the fetus. Though the distance between the catheter basket and the fetal chest wall was short at first, the catheter was gradually displaced into the amniotic cavity because the distance between the catheter basket on the thoracic cavity side and the fetal chest wall was long in the first and second TAS, similar to the issues observed in previous studies [2-4]. A practical approach to prevent such displacement is to use a catheter with a reduced length. We were able to prevent catheter displacement by using a 50 mm catheter instead of a 60 mm catheter, which reduced the distance between the catheter basket on the amniotic fluid cavity side and the fetal thoracic cavity side, thereby minimizing displacement risks. This approach aligns with the recommendations of Takahashi et al., who emphasized the importance of tailoring catheter length and placement based on the anatomical dimensions of the fetal chest to ensure effective shunting and minimize complications [3]. However, we must consider the risk of leaving either end of the catheter embedded into the oedematous fetal skin, especially in hydrops cases.

## Conclusions

To prevent catheter displacement due to fetal movement, it is crucial to adjust the length and placement position of the catheter based on the thickness of the fetal chest wall. In cases with hydrops, consideration should be given to the risk of leaving either end of the catheter embedded in the edematous fetal skin.
